# Finite Element Analysis of Ultra-Short, Extra-Short, and Conventional Dental Implants in Fibula Free Flap Mandibular Reconstruction

**DOI:** 10.3390/bioengineering13091052

**Published:** 2026-09-10

**Authors:** Mario Ceddia, Luciano Lamberti, Bartolomeo Trentadue

**Affiliations:** Department of Mechanics, Mathematics and Management, Polytechnic University of Bari, 70125 Bari, Italy; luciano.lamberti@poliba.it (L.L.); bartolomeo.trentadue@poliba.it (B.T.)

**Keywords:** fibula free flap, dental implants, short implants, finite element analysis, mandibular reconstruction

## Abstract

Dental rehabilitation of fibula free flap mandibular reconstructions can be limited by the reduced vertical bone height available for implant placement. This study evaluated the biomechanical influence of ultra-short, extra-short, and conventional implants in a reconstructed fibula. Methods: A patient-specific finite element model of a fibula-reconstructed mandible was developed. Three splinted implants with a diameter of 4 mm and lengths of 3, 5, or 12 mm were compared under postoperative unilateral molar loading. Von Mises stresses were evaluated in the implants, fibula, residual mandible, and fixation plates, while equivalent elastic strain was assessed in peri-implant bone. Results: Implant length had minimal influence on implant stress (36.8–37.1 MPa) and fibular cortical bone stress (52.5–53.1 MPa). The 5 mm implants produced the lowest peri-implant strains and the lowest posterior reconstruction-plate stress (71.3 MPa), compared with 75.4 MPa for 3 mm and 107.8 MPa for 12 mm implants. The 12 mm implants generated greater apical strain concentrations, exceeding the adopted mechanobiological threshold around the central and mesial implants. Conclusions: Within the limitations of this study and under the simulated immediate postoperative loading condition, shorter implants, particularly 5 mm implants, showed a favorable biomechanical response.

## 1. Introduction

Mandibular reconstruction is essential for restoring mastication, speech, facial aesthetics, and patient identity. Among the available reconstructive options, the fibula free flap is widely used for segmental and complex bone defects because of its versatility, clinical reliability, and favorable bone quality for implant integration [1,2,3].

Although fibula free flap reconstruction reliably restores mandibular continuity, improving masticatory function remains a major clinical challenge [3]. In the study by Romero et al. [4], chewing had the lowest mean score (57.1) of all the domains assessed using a health-related quality of life (HRQoL) questionnaire. Furthermore, nine of the thirteen patients who provided free-text comments expressed a desire for dental rehabilitation after mandibular reconstruction, primarily to enhance mastication and aesthetics.

Functional dental rehabilitation after fibula free flap reconstruction has traditionally required staged surgical and prosthetic protocols, with overall treatment times of 6–18 months [5,6]. To overcome these limitations, digitally driven workflows have been developed to combine virtual planning, fibula free flap reconstruction, immediate implant placement, and provisional fixed prosthetic rehabilitation within a single surgical procedure [7].

This approach was subsequently refined and extended to include both mandibular and maxillary reconstruction under the “Jaw in a Day” (JIAD) concept. The aim of JIAD is to promote the early restoration of facial form, oral function and aesthetics by integrating resection, reconstruction, implant placement and immediate provisional prosthetic rehabilitation into a single surgical procedure [8,9,10].

Since its introduction, case reports and technical studies have demonstrated the feasibility and procedural accuracy of the JIAD approach, with favorable short-term clinical outcomes [9,10,11,12].

Nevertheless, delayed implant placement remains common because it allows flap healing, soft-tissue maturation, and definitive prosthetic planning before implant insertion [13]. Immediate implant placement offers several advantages, including direct surgical access to the fibular bone, improved control of prosthetically driven implant positioning relative to the opposing dentition, and a shorter time to dental rehabilitation [13,14]. However, delayed implant placement is still frequently performed in clinical practice to reduce the risk of damaging the vascularized bone.

In oncologic patients, implant timing is further influenced by radiotherapy, which may increase the risk of complications, including osteoradionecrosis [15]. Panchal et al. [16] reported a trend toward higher implant survival when implants were placed before radiotherapy compared with placement after radiotherapy, with survival rates of 88.9% and 83.4%, respectively.

Favorable outcomes for immediate implant placement were also reported by Kim et al. [17], who observed a 100% implant survival rate for implants placed according to the JIAD protocol during fibula free flap reconstruction.

From an implant-prosthetic perspective, the fibula provides favorable bone quality for osseointegration and functional rehabilitation. However, its triangular cross-section and reduced height compared with the native mandible may create a vertical discrepancy between the superior border of the fibular graft and the opposing dentition, particularly when the graft is aligned with the inferior mandibular border [18,19,20].

The bicortical anatomy of the fibula may facilitate high primary implant stability, and engagement of both cortices is therefore frequently adopted during implant rehabilitation of fibular grafts [20,21]. However, bicortical implant placement requires careful evaluation of the residual bone geometry. A clinical report described an imminent pathological fracture in a fibular neomandible affected by severe peri-implant bone loss around a through-and-through implant, with a particularly high fracture risk during implant removal [22]. In these cases, short, extra-short, and ultra-short implants may represent a suitable alternative because they require less vertical bone height and may reduce the need for additional bone augmentation procedures [23,24].

Hocková et al. [25] observed 100% survival of short and extra-short implants placed in osseous microvascular free flaps, with an implant success probability of 91.0%. Nevertheless, earlier studies suggested that short implants might be associated with a higher risk of failure, particularly in irradiated patients, and consequently recommended the use of surface-treated implants with a length of approximately 10 mm for the rehabilitation of vascularized fibula grafts [26,27,28].

These findings suggest that implant length may play an important role not only from a clinical perspective, but also from a biomechanical standpoint. Indeed, implant success depends not only on biological factors, but also on load-transfer mechanisms from the prosthetic suprastructure to the peri-implant bone [29,30,31].

In this context, finite element analysis has been used to investigate the effects of implant position [32], implant number [33], prosthetic splinting [34], and implant geometry [35,36] on stress transfer within reconstructed mandibles and peri-implant bone.

To the best of our knowledge, the combined effects of implant length and prosthetic splinting have not yet been investigated in a patient-specific model of a fibula-reconstructed mandible. Therefore, this study used finite element analysis to compare ultra-short, extra-short, and conventional splinted implants and assess whether reduced-length configurations may provide a biomechanically favorable alternative to conventional implants.

## 2. Materials and Methods

FE models were developed based on the study by Ruf et al. [37], in which a virtually reconstructed mandible was modelled after segmental resection and subsequent reconstruction using a free fibular flap. This model was derived from the preoperative CT scans of a 57-year-old female patient with oral squamous cell carcinoma affecting the right floor of the mouth with bone invasion. The mandibular resection extended from the mandibular angle to the right canine region. The bone geometries were exported as surface models (.stl) and imported into Autodesk Inventor Professional 2024 (Autodesk, Inc., San Francisco, CA, USA), where the preliminary mesh and assembly of the model components was performed.

### 2.1. Fixation System and Dental Implant Placement

**Figure 1:**
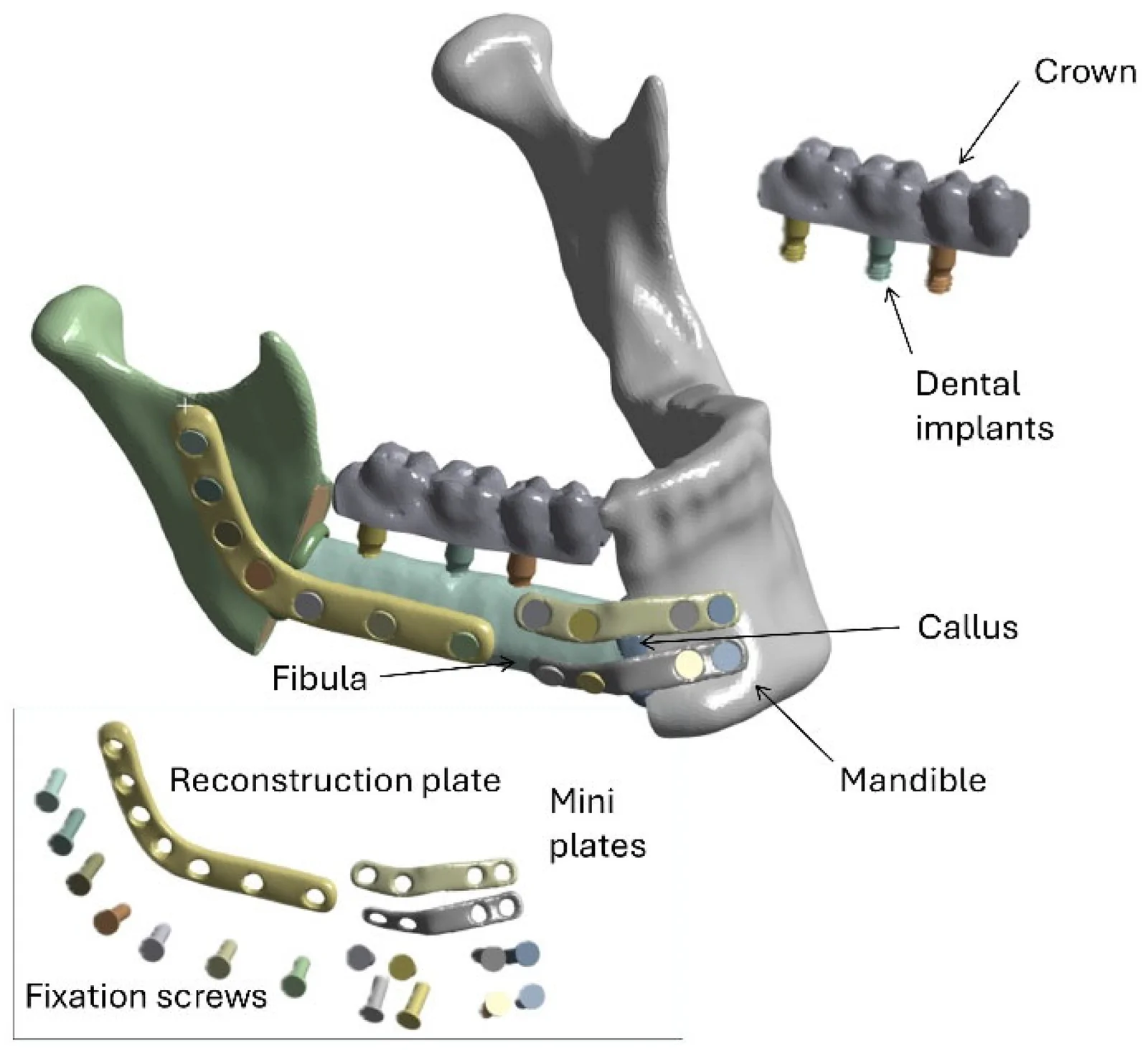
Three-dimensional representation of the FEM model analyzed in this study, including the residual mandible, reconstructed fibula, fixation system, and dental implants.

In the mandibular angle region, a reconstruction plate 2 mm thick was used and fixed in place with seven bicortical screws (four in the residual mandible and three in the fibula). These screws had a diameter of 2.0 mm and lengths ranging from 7 to 8 mm. The two miniplates were fixed with monocortical screws, each with a diameter of 2.0 mm and a length of 7 mm (see Figure 1). The plates were designed using Geomagic Freeform Plus v2021.0.56 software (3D Systems, Rock Hill, SC, USA), and the screws used to fix the reconstruction plate and miniplates were modelled with simplified geometry to reduce computational costs, as they were not the primary focus of this study [37].

Three implant configurations were generated. Each configuration included three implants with a diameter of 4 mm and an identical length of either 3, 5, or 12 mm inserted into the fibula at the crest level (AoN Implants Srl, Grisignano di Zocco, Italy).

**Figure 2:**
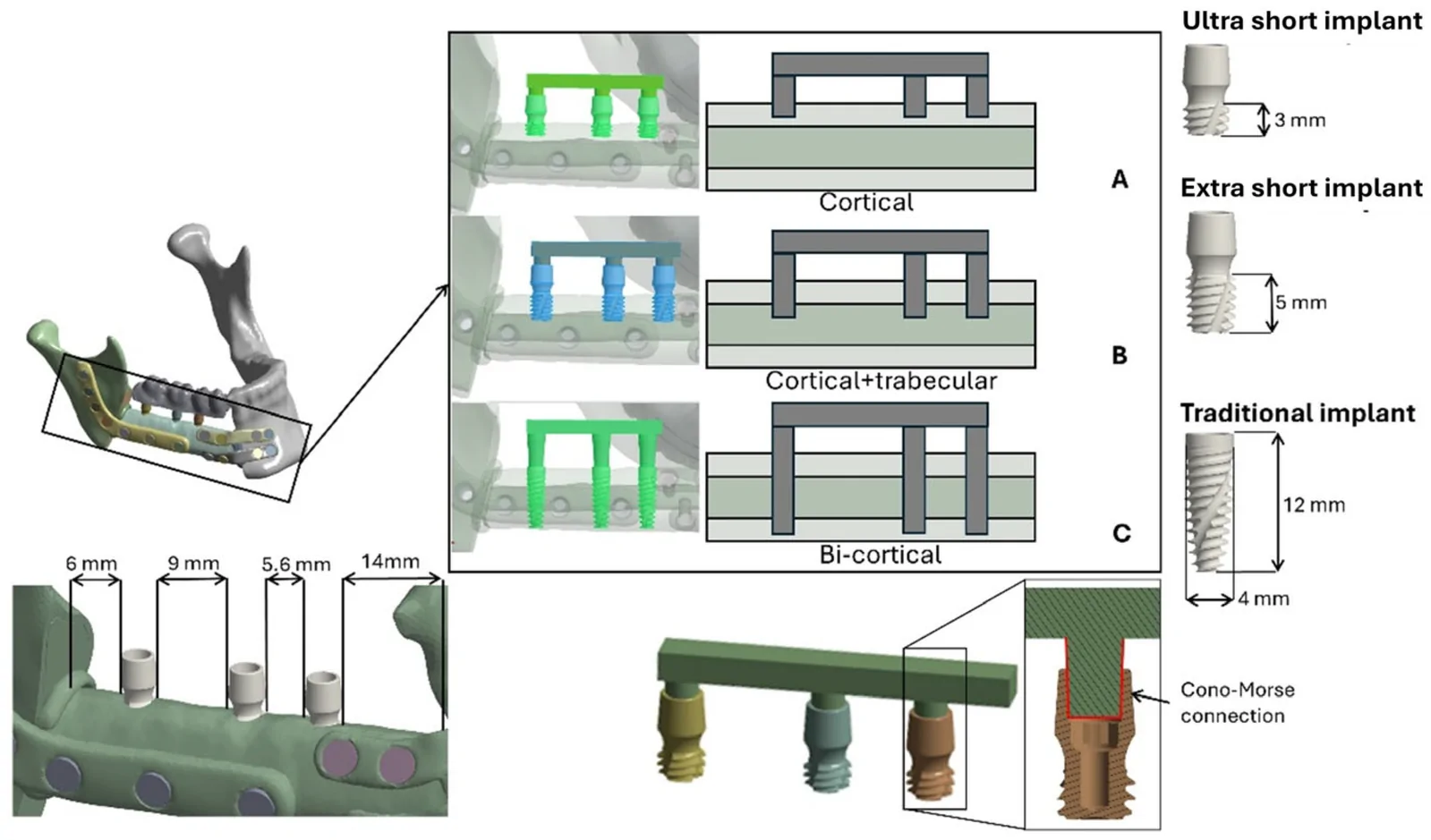
Scheme of implant placement in the reconstructed fibula, with three implants connected by a titanium bar.

The posterior and anterior implants were positioned 6 mm and 14 mm from the corresponding fibular osteotomy edges, respectively. The three implants were splinted together with a titanium bar and a distance of more than 3 mm was maintained between them. This was in accordance with clinical recommendations for the placement of multiple implants, as well as rehabilitation protocols for free fibular flap implant placement [38,39,40,41] (Figure 2).

### 2.2. Meshing

The previously created geometrical model was converted into a solid format (.stp) using Ansys SpaceClaim 2024 R2 (ANSYS, Inc., Canonsburg, PA, USA). The model was then meshed in Ansys Workbench 2024 R2 (ANSYS, Inc., Canonsburg, PA, USA) using second-order quadratic tetrahedral finite elements.

Mesh sizes of 0.2 mm, 0.25 mm, and 1.0 mm were adopted for the bone callus, fixation plates, and screws, respectively. A dedicated mesh convergence analysis was performed for the dental implants by varying the element size from 0.8 mm to 0.5 mm and evaluating the maximum von Mises stress on the implants for each configuration of the mesh.

The 0.5 mm mesh size resulted in a variation in Von Mises stress less than 1% between two consecutive iterations and was therefore selected for the discretization of the dental implants.

For the final model, using a 0.5 mm element size for the dental implants, the finite element mesh consisted of approximately 714,539 nodes and 416,209 s-order tetrahedral elements.

### 2.3. Boundary Conditions

Several studies have reported that contralateral molar occlusion results in high levels of mechanical stress and may therefore be a critical condition for mandibular clenching [42,43,44]. Accordingly, unilateral clenching on molar regions was selected in the present study as the critical loading scenario.

Two occlusal configurations were simulated by constraining vertical displacement at the first molar region of either the healthy side or the implant-supported reconstructed side.

**Figure 3:**
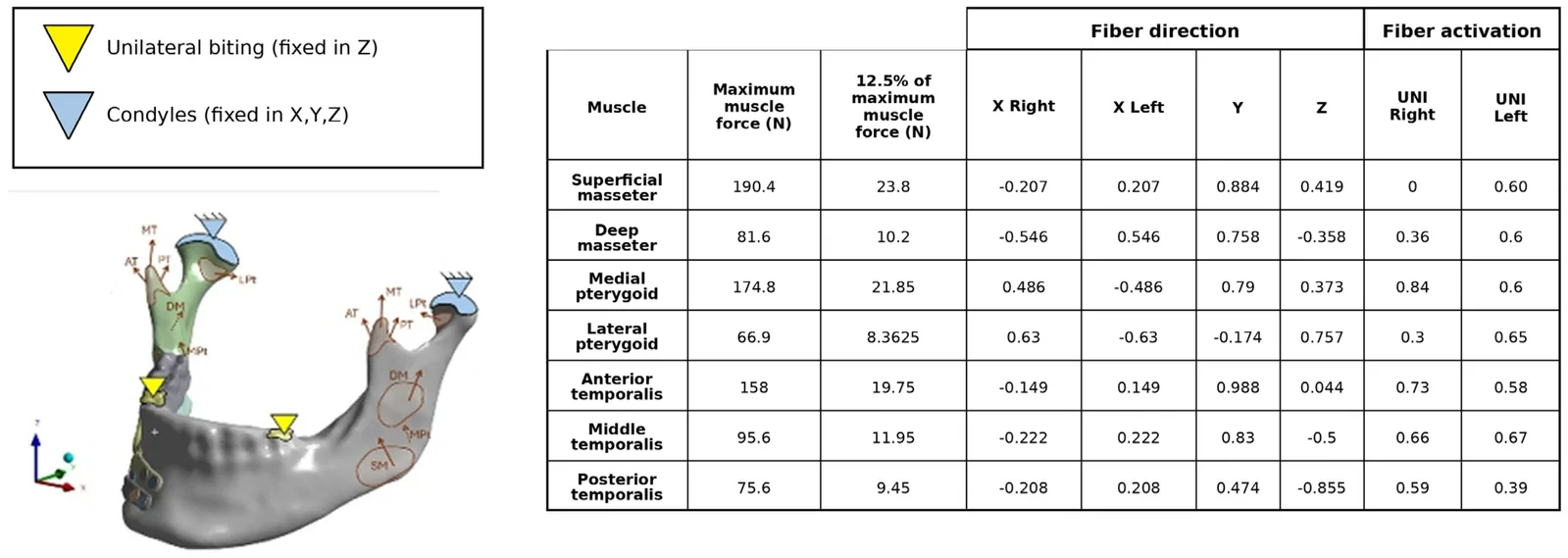
Muscle forces, fiber directions, and activation patterns of the masticatory muscle groups used to simulate right unilateral clenching on the dental implant-supported suprastructure placed in the reconstructed fibula.

For the loading condition on the healthy side, muscle forces were derived from the study by Ruf et al. [37]. The muscle activation pattern and force vector directions described by Korioth et al. [45] were then adapted to simulate clenching on the reconstructed, implant-supported side for the loading condition applied to the dental implants (Figure 3).

To reproduce a postoperative scenario, the maximum muscle forces were reduced to 12.5% of the physiological values, resulting in a unilateral bite force of 40 N [46].

This loading condition was intended to represent the immediate postoperative phase and should not be considered representative of later-stage functional mastication after recovery of masticatory muscle activity. In addition, on the side ipsilateral to the defect, the superficial masseter was considered detached, while partial detachment of the deep masseter was simulated by reducing its activation by 50%.

The healthy-side loading condition was simulated once and used as a common reference for comparison with the three implant-supported loading configurations, since implant length was not treated as a variable for this reference condition.

During the power stroke of mastication, the condyles were assumed to be locked within the glenoid fossae and were therefore constrained in all six degrees of freedom.

The postoperative loading conditions were reproduced by simulating the activation of the main masticatory muscles, including the superficial masseter (SM), deep masseter (DM), anterior temporalis (AT), middle temporalis (MT), posterior temporalis (PT), medial pterygoid (MPt), and inferior lateral pterygoid (LPt).

Bonded contact conditions were used to model the fixation screw-bone and plate-screw interfaces. For immediately loaded implant models, contact interfaces (non-osseointegration) between the implant and bone were simulated.

In accordance with Tang et al. [47], frictional contact was assigned at the implant–bone interfaces, with friction coefficients of 0.65 for cortical bone–implant contact and 0.77 for cancellous bone–implant contact. A friction coefficient of 0.3 was assigned to the titanium–titanium interfaces.

### 2.4. Material Properties

**Figure 4:**
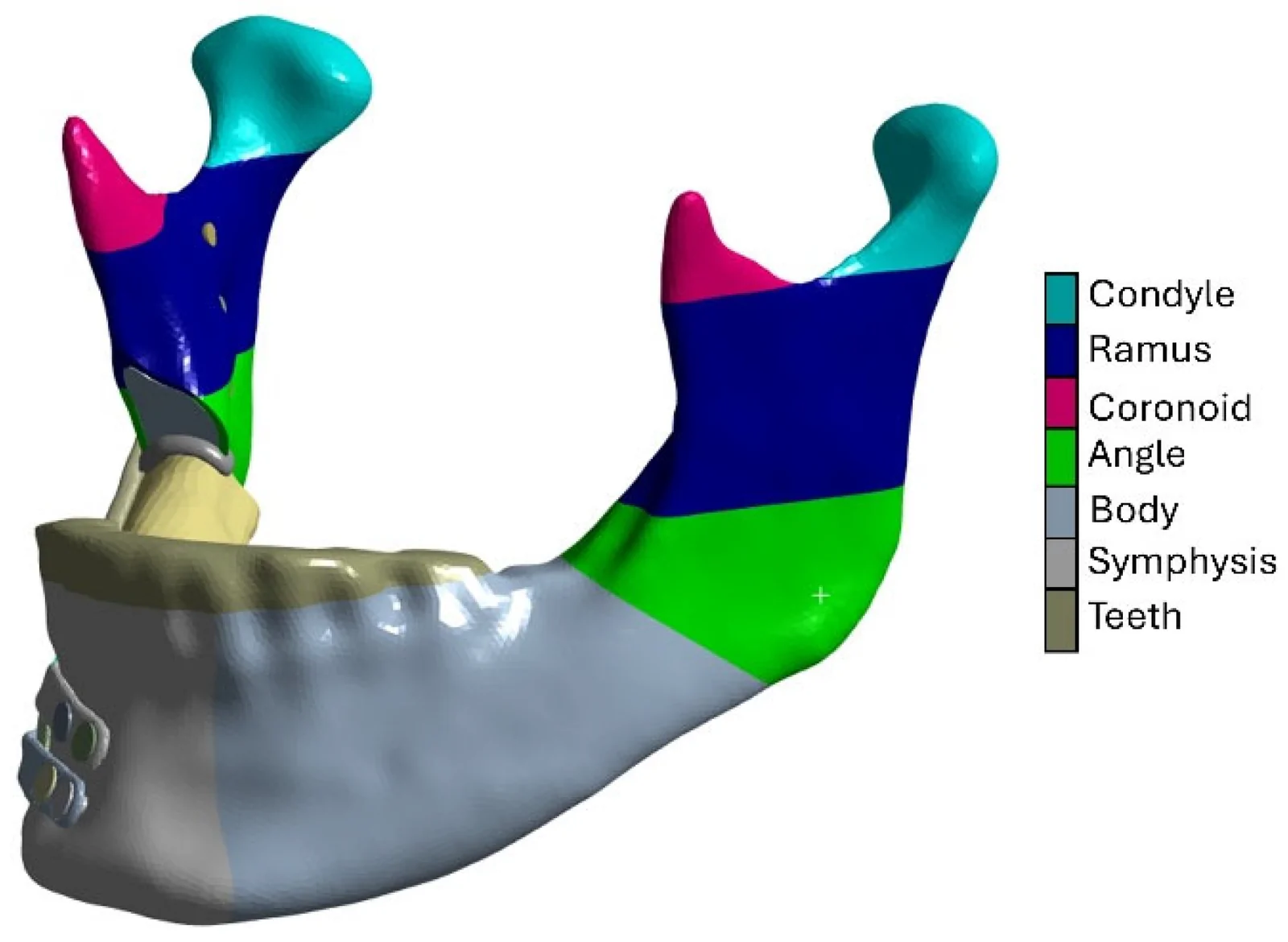
Subdivision of the cortical bone into six anatomical regions, each assigned specific orthotropic material properties.

The cortical bone of the mandible and fibula was modelled as a homogeneous, orthotropic, linearly elastic material. According to the classification proposed by Schwartz et al. [48], the mandibular cortical volume was divided into six anatomical regions: symphysis, body, angle, ramus, condyle, and coronoid (Figure 4). This subdivision was performed using the Split command in SpaceClaim. Region-specific orthotropic elastic properties were then assigned to the cortical finite elements through local coordinate systems [43,48].

The anisotropic Young’s and shear moduli of the fibular cortical bone were assigned according to Lefèvre et al. [49], while the Poisson’s ratios were taken from analogue tibial regions reported by Rho et al. [50].

The trabecular bone of the mandible and fibula, teeth, and prosthetic components, including screws, dental implants, and fixation plates, were modelled as isotropic, homogeneous, linearly elastic materials [45,51,52]. For Ti6Al4V, a yield strength of 830 MPa was used as the reference threshold for assessing potential material failure [53]. For cortical bone, a tensile strength of 133 MPa was considered as the reference threshold [54].

The bone callus was assigned isotropic material properties representative of granulation tissue to replicate the immediate post-operative condition, with a Young’s modulus of 0.2 MPa and a Poisson’s ratio of 0.167 [55]. All mechanical properties assigned to the mandibular bone, fibular bone, bone callus, teeth, and prosthetic components are reported in Table 1.

### 2.5. Output Evaluation

Equivalent von Mises stress was used to evaluate the mechanical response of the regions of interest, which included the fixation plates, fibula, dental implants and residual mandible. The peak von Mises stress values for each component were considered indicators of potential component failure risk according to the relevant strength criteria: yield strength for the prosthetic components and tensile strength for bone tissue. The upper 0.01% of stress values was excluded, and the highest remaining value was reported as the filtered maximum stress.

To minimize the effect of possible numerical discontinuities at the edges of the elements, stress values were extracted from the element integration points and visualized in ANSYS 2024 R2 (ANSYS, Inc., Canonsburg, PA, USA) using the average nodal results. This reduces the effect of numerical discontinuities between adjacent elements compared to unaveraged contour plots.

Additionally, a 1 mm thick peri-implant region of interest was defined around each implant, and the equivalent elastic strain was extracted from the bone elements contained within this region. The maximum equivalent elastic strain within this region was extracted and interpreted according to previously established mechanobiological thresholds for bone maintenance, formation, and resorption [56,57].

## 3. Results

### 3.1. Stress on Fixation Plates

**Figure 5:**
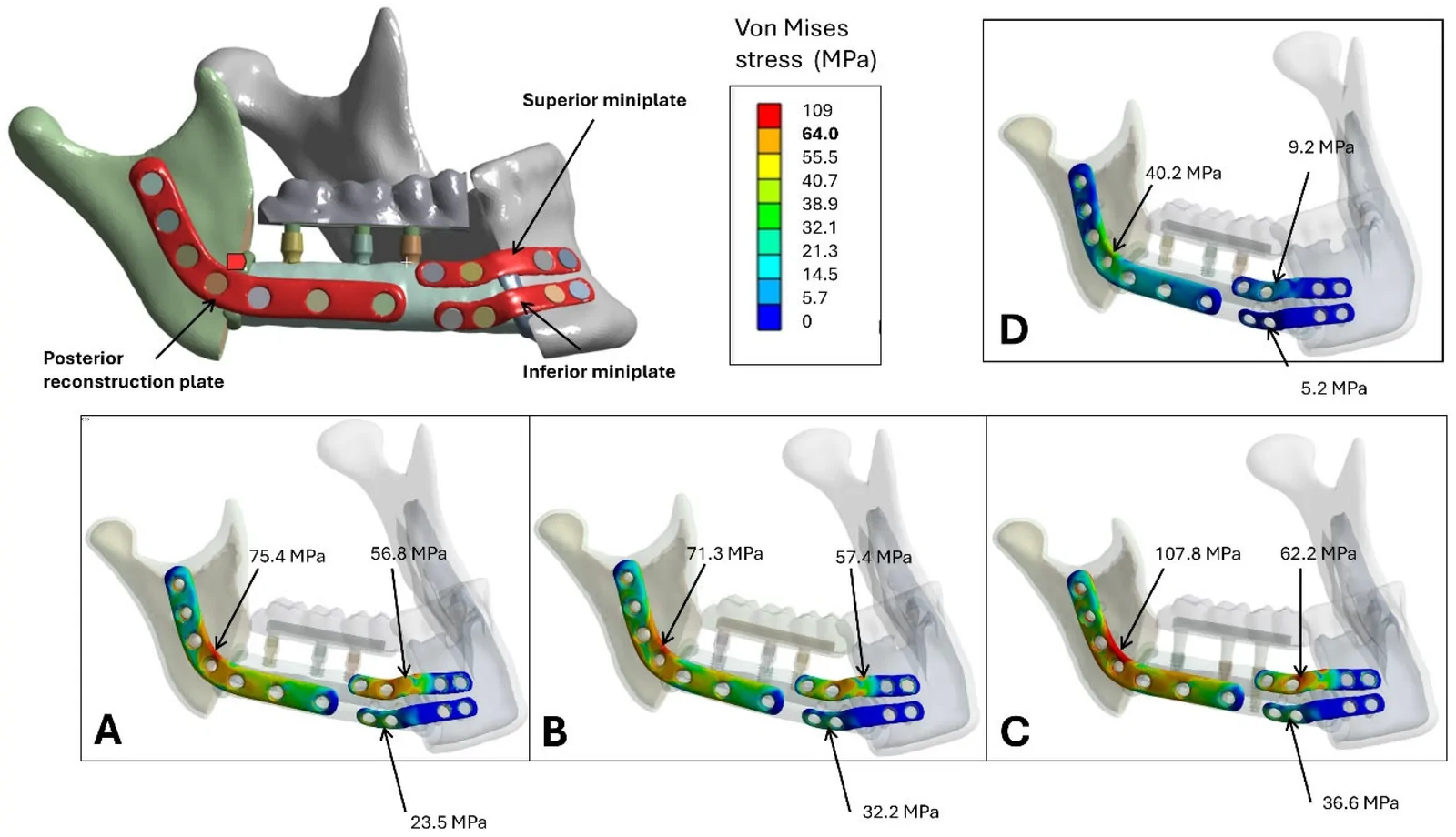
Von Mises stress distribution in the fixation plates under molar loading on the implant-supported prosthesis with implant lengths of 3 mm (**A**), 5 mm (**B**), and 12 mm (**C**), under loading on the healthy side (**D**). Stress values are expressed in MPa.

The von Mises stress distribution in the fixation plates under molar loading applied to the implant-supported prosthetic crowns on the reconstructed right side is shown in Figure 5. This figure shows the distribution for implant lengths of 3 mm (Figure 5A), 5 mm (Figure 5B), and 12 mm (Figure 5C). The same distribution is also shown for molar loading on the healthy left side (Figure 5D). Loading on the healthy side resulted in lower stresses in the posterior reconstruction plate, as well as in the superior and inferior miniplates, than loading on the implant-supported prosthesis.

With loading on the prosthetic side, the highest stress, 107.8 MPa, was observed with 12 mm implants, concentrated in the mandibular angle region of the posterior reconstruction plate. This was higher than the stresses observed with 5 and 3 mm implants, which were 71.3 and 75.4 MPa, respectively. The anterior miniplates exhibited lower stresses overall. Of these, the superior miniplate had the highest values, reaching 56.8 MPa, 57.4 MPa, and 62.2 MPa for 3, 5, and 12 mm implant lengths, respectively.

**Figure 6:**
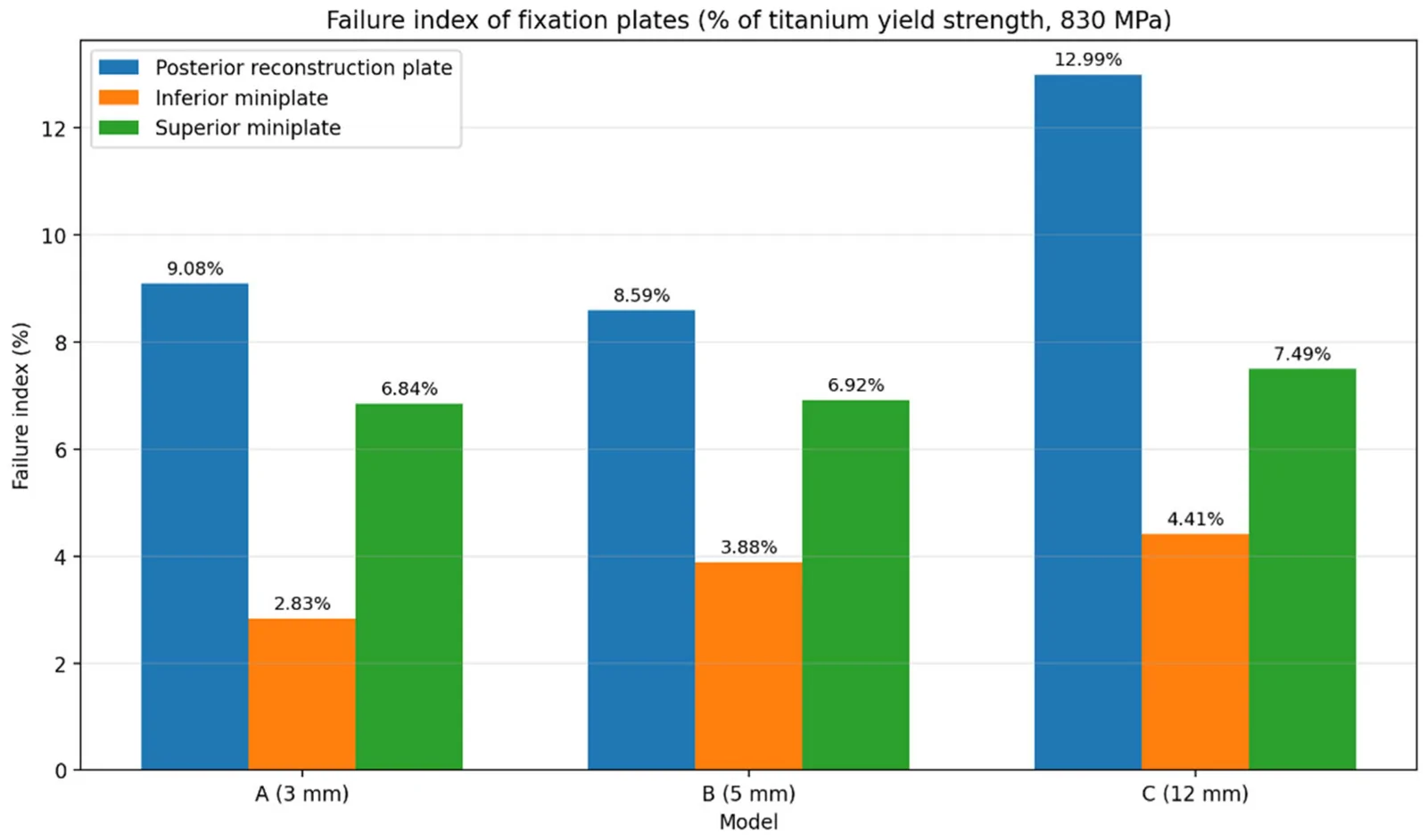
Failure index of the posterior reconstruction plate and anterior miniplates for implant lengths of 3 mm (Model (**A**)), 5 mm (Model (**B**)), and 12 mm (Model (**C**)).

The von Mises stresses in the fixation plates shown in Figure 5 were evaluated with respect to the titanium yield strength. For each plate, a failure index was calculated as the ratio of the maximum von Mises stress to the material yield strength and expressed as a percentage, as reported in Figure 6.

The highest failure index was observed in the posterior reconstruction plate of Model C, reaching 12.99%, compared with 8.59% in Model B and 9.08% in Model A. For both anterior miniplates, the failure index progressively increased with implant length.

### 3.2. Stress on Fibula Bone

**Figure 7:**
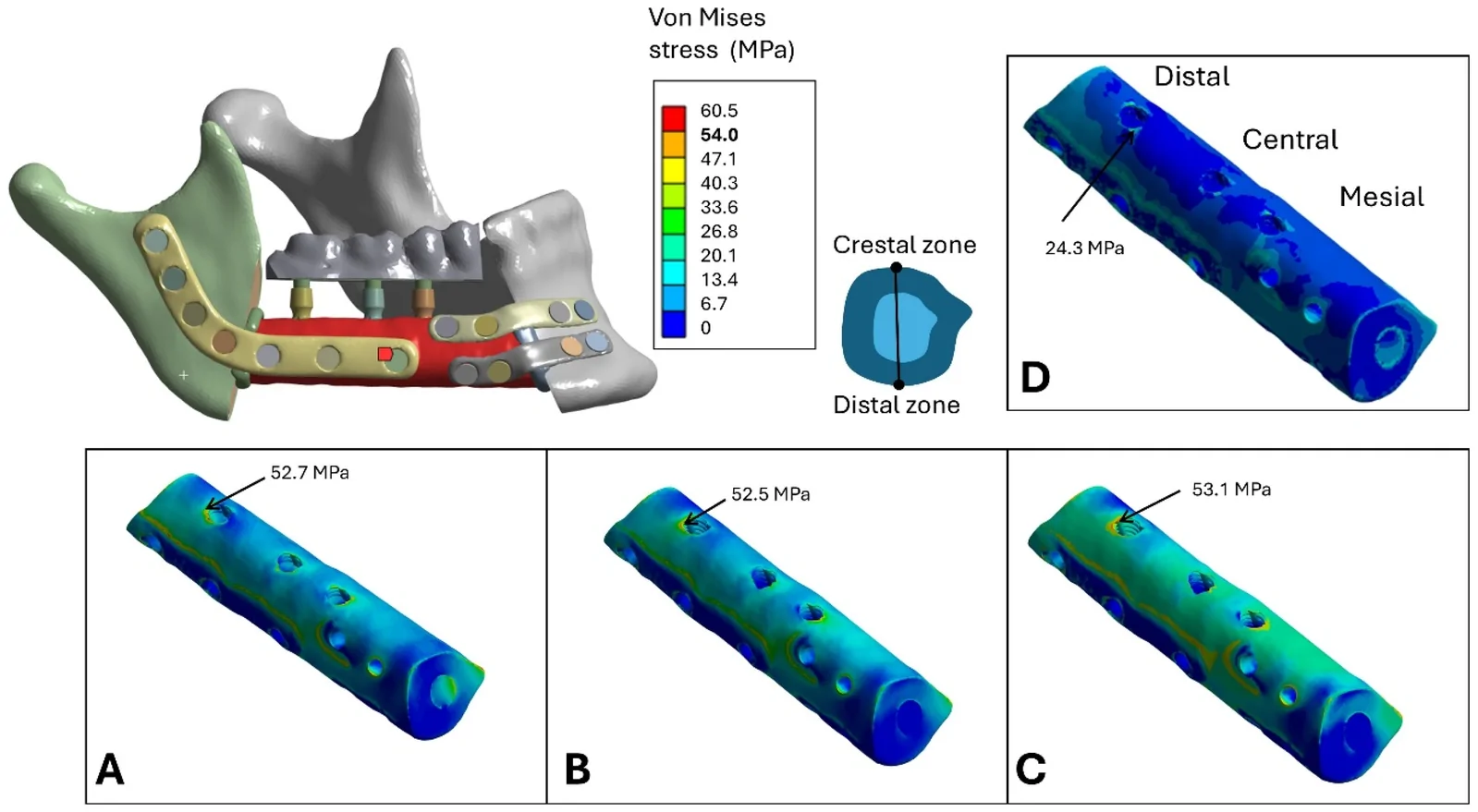
Von Mises stress distribution in the cortical bone of the fibular graft under molar loading on the implant-supported prosthesis with implant lengths of 3 mm (**A**), 5 mm (**B**), and 12 mm (**C**), and under loading on the healthy side (**D**).

The von Mises stress distribution in the cortical bone of the fibular graft is shown in Figure 7. In all configurations, the maximum stress was localized at the crestal zone of the distal implant osteotomy. Unilateral clenching on the healthy side of the mandible produced the lowest maximum stress, reaching 24.3 MPa (Figure 7D). Under loading of the implant-supported prosthesis, comparable maximum stresses of 52.7 MPa and 52.5 MPa were observed for implant lengths of 3 mm and 5 mm, respectively (Figure 7A,B). A slight increase to 53.1 MPa was observed with the 12 mm implants (Figure 7C).

### 3.3. Stress on Mandible

**Figure 8:**
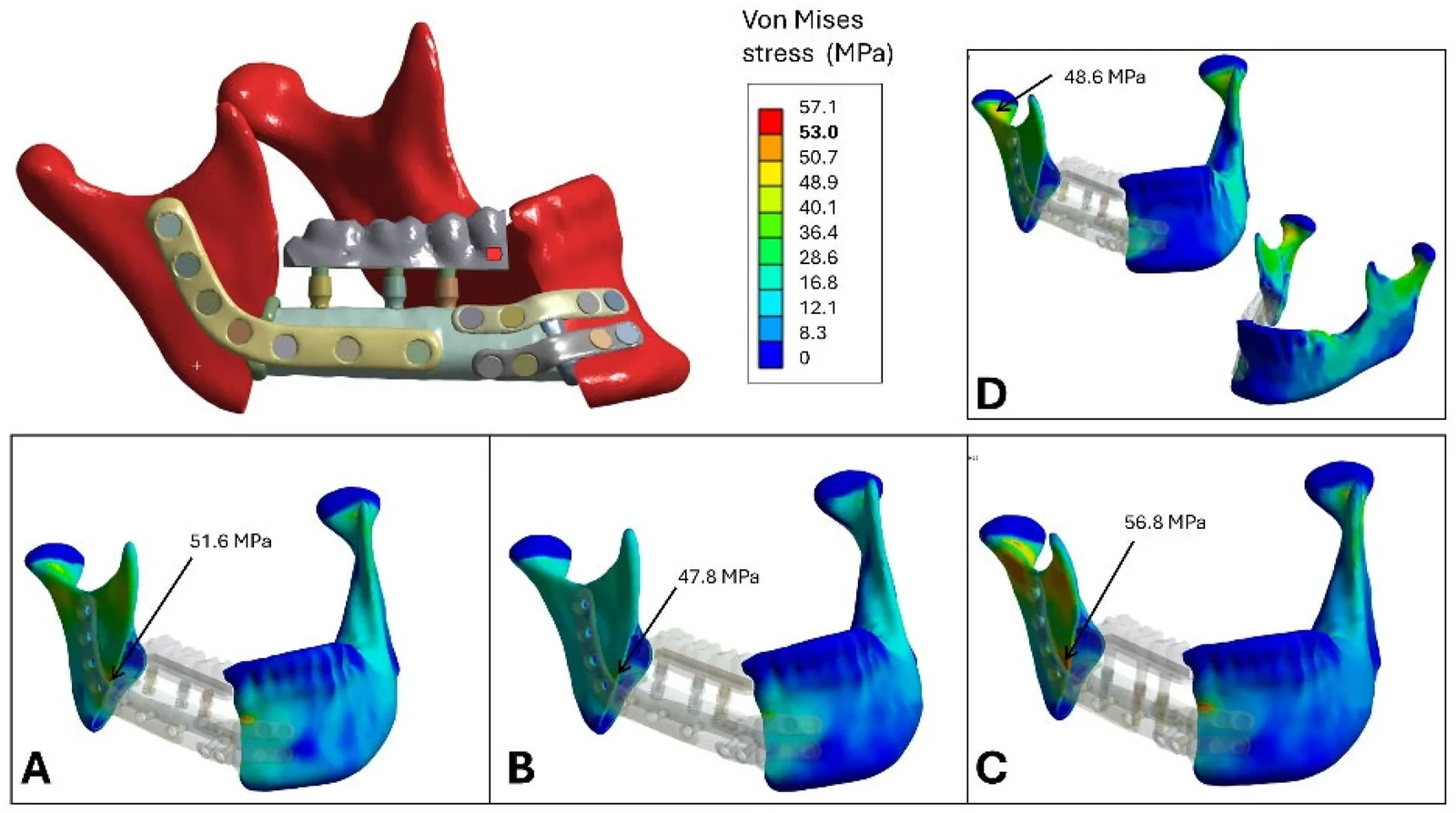
Von Mises stress distribution in the residual mandible under molar loading on the implant-supported prosthesis with implant lengths of 3 mm (**A**), 5 mm (**B**), and 12 mm (**C**), under loading on the healthy side (**D**).

The von Mises stress distribution in the residual mandible showed distinct stress concentration patterns depending on the loading condition. Under molar clenching on the healthy side, the maximum stress was localized at the condyle–ramus transition on the reconstructed side, reaching 48.6 MPa (Figure 8D). An additional stress concentration of 32.4 MPa was observed near the loaded first molar, at the tooth–bone transition.

When the load was applied to the implant-supported prosthesis, the peak stress occurred at the interface between the posterior reconstruction plate and the mandibular ramus. The highest value was recorded with 12 mm implants, reaching 56.8 MPa (Figure 8C), compared with 51.6 MPa and 47.8 MPa for the 3 mm and 5 mm implants, respectively (Figure 8A,B). Further stress concentrations were also observed at the contact region between the superior anterior miniplate and the mandibular body.

### 3.4. Stress on Dental Implants

**Figure 9:**
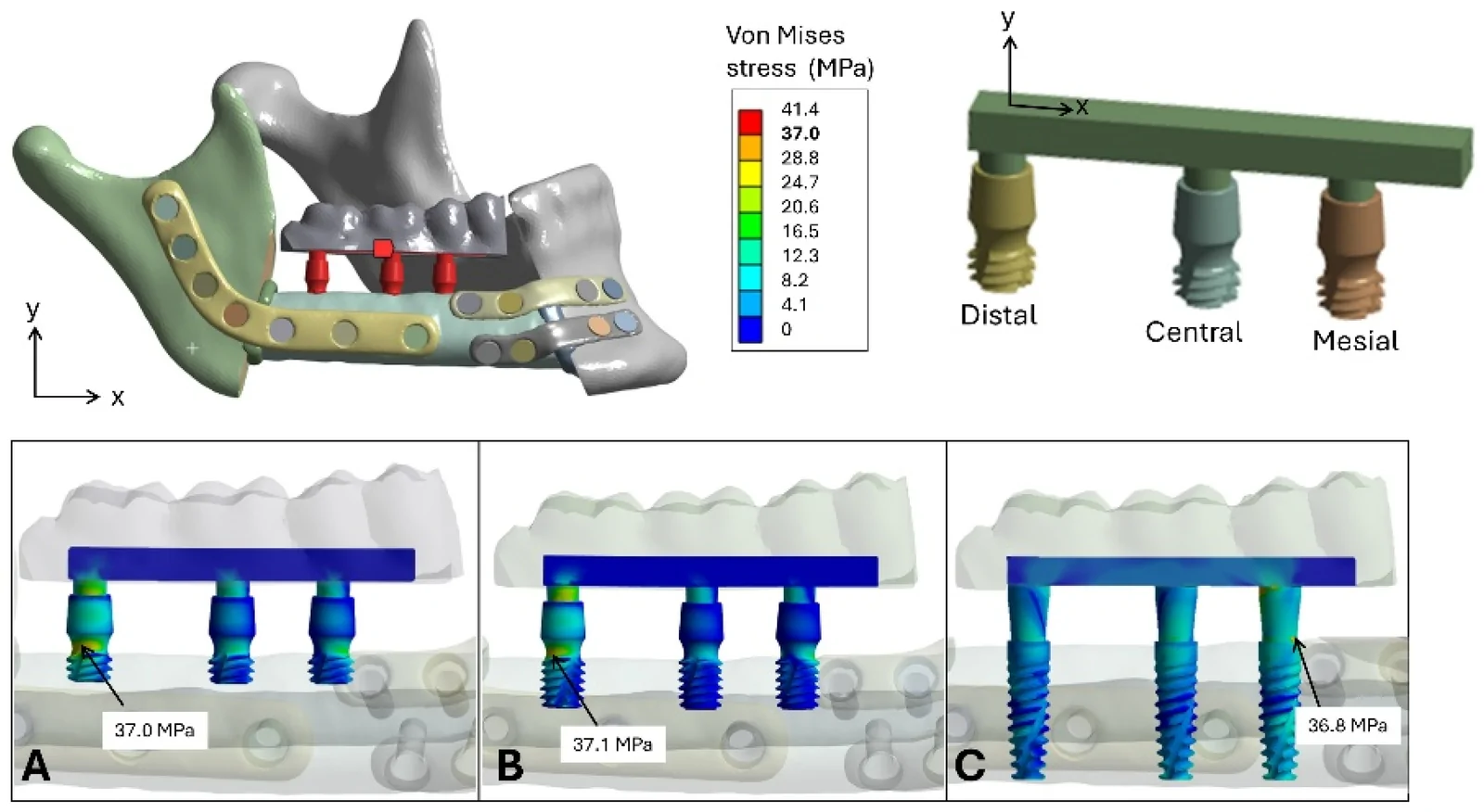
Von Mises stress distribution in the three splinted implants under molar loading on the implant-supported prosthesis for implant lengths of 3 mm (**A**), 5 mm (**B**), and 12 mm (**C**). Distal, central, and mesial implant positions are indicated.

The maximum von Mises stress showed only minimal variation among the three implant lengths, ranging from 36.8 to 37.1 MPa. For the 3 mm and 5 mm lengths (Figure 9A,B), the peak stress was localized at the neck of the distal implant and reached 37.0 and 37.1 MPa, respectively. For the 12 mm implant (Figure 9C), the peak shifted to the implant-abutment connection of the mesial implant with a slightly lower value of 36.8 MPa.

### 3.5. Strain at the Bone-Implant Interface

**Figure 10:**
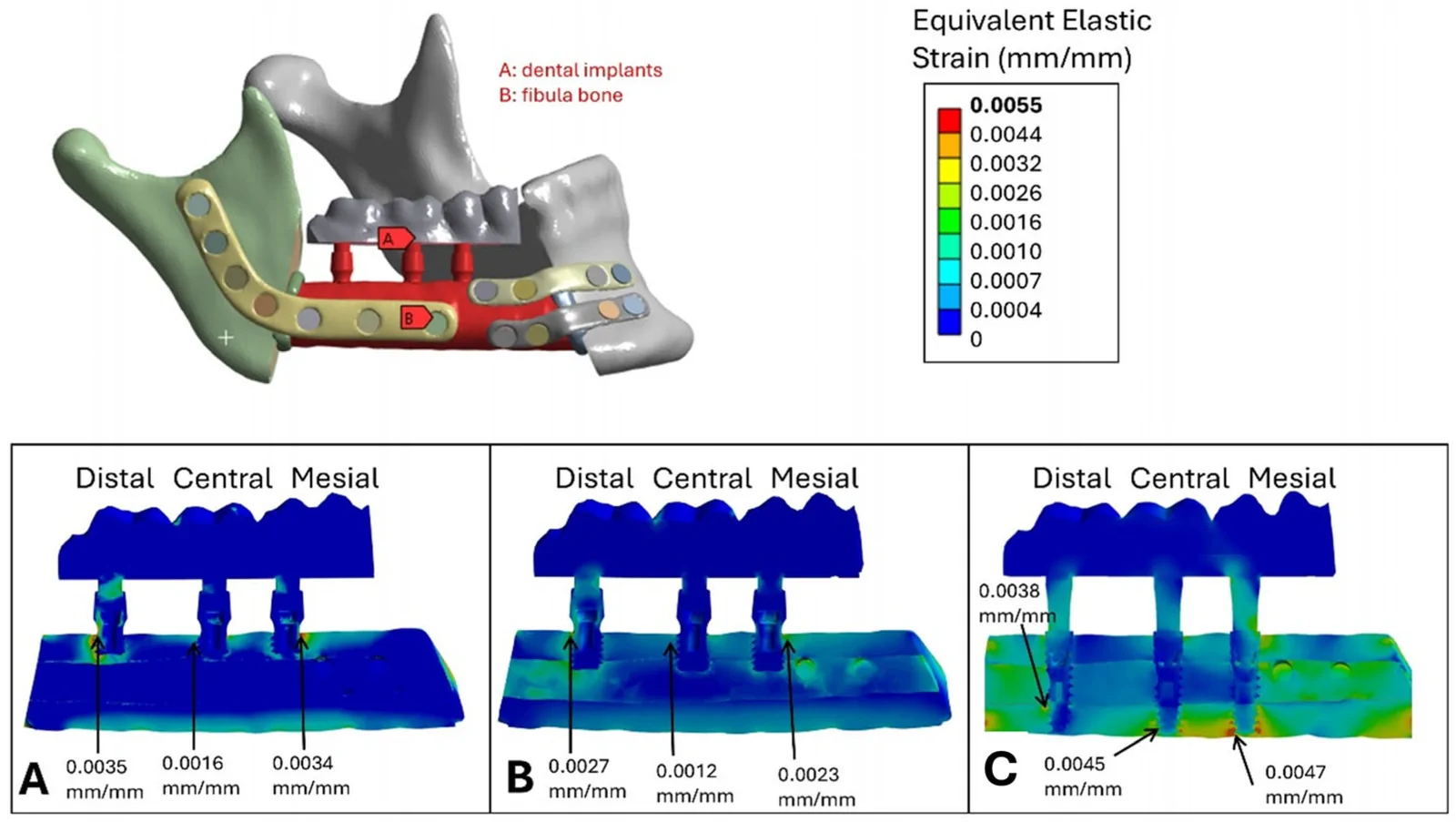
Equivalent elastic strain distribution in the peri-implant fibular bone for implant lengths of 3 mm (**A**), 5 mm (**B**), and 12 mm (**C**). Strain values were evaluated within a 1 mm thick peri-implant region surrounding the implant surface and are expressed in mm/mm.

The equivalent elastic strain distribution in the peri-implant fibular bone varied according to implant length. For the 3 mm and 5 mm configurations (Figure 10A,B), the highest strain values were concentrated in the crestal region around the distal and mesial implants, whereas lower values were observed around the central implant. In Model A, the maximum strains were 0.0035 mm/mm, 0.0016 mm/mm, and 0.0034 mm/mm around the distal, central, and mesial implants, respectively. In Model B, the corresponding values decreased to 0.0027 mm/mm, 0.0012 mm/mm, and 0.0023 mm/mm.

With 12 mm implants, strain increased around all three implants, and the main concentrations shifted toward the apical regions. Maximum values of 0.0038 mm/mm, 0.0045 mm/mm, and 0.0047 mm/mm were recorded around the distal, central, and mesial implants, respectively. The values observed around the central and mesial implants exceeded the adopted mechanobiological threshold of 0.0040 mm/mm [56], suggesting regions of elevated mechanical stimulus that may be associated with an increased risk of bone microdamage.

## 4. Discussion

In this patient-specific FE model, reducing implant length from 12 mm to 5 or 3 mm under immediately loaded, splinted conditions had only a limited influence on peak von Mises stress in the implants and fibular cortical bone. Implant stresses ranged from 36.8 to 37.1 MPa, while peak fibular cortical bone stresses ranged from 52.5 to 53.1 MPa. In contrast, implant length was associated with more pronounced differences in peri-implant strain distribution and load transfer to the osteosynthesis system. Within the present model, the 5 mm configuration showed the lowest peri-implant strains, whereas the 12 mm implants were associated with a shift in strain toward the apical regions, reaching 0.0045 and 0.0047 around the central and mesial implants, respectively, and exceeding the adopted mechanostat threshold of 0.004. The 12 mm configuration also generated the highest posterior plate stress (107.8 MPa), compared with 75.4 and 71.3 MPa for the 3 and 5 mm configurations, respectively. Nevertheless, all plate stresses remained well below the Ti6Al4V yield strength. These findings suggest that, under the specific anatomical, material, and loading conditions considered in this patient-specific model, shorter implants may provide a mechanical response comparable to that of longer implants, while altering peri-implant strain distribution and load transfer. Accordingly, increasing implant length did not appear to provide a clear biomechanical advantage in the present model. However, because the analysis was based on a single patient-specific geometry, these findings should be interpreted with caution and confirmed in future studies including a broader range of anatomical and clinical conditions. Walsh et al. [58] similarly reported a non-linear relationship between implant dimensions and fibular stress, with stress decreasing to an optimal size and subsequently increasing as implant volume became excessive; implant length also had a weaker effect than diameter. This is consistent with the lower peri-implant strains observed for the 5 mm configuration under the specific 40-N postoperative loading condition investigated here. Conversely, Wu et al. [59] reported lower fibular stresses and micromotion with longer, bicortically engaged implants than with a shorter monocortical implant. This difference likely reflects model design: Wu et al. [59] used a simplified single-implant fibula, whereas the present patient-specific model includes splinting, fixation system and muscle-derived loading. Thus, the local benefit of bicortical engagement should not be interpreted as evidence that greater implant length is always globally advantageous. Similarly, Yukawa et al. [60] showed that bicortical fixation reduced peri-implant collar stress under centered loading but also demonstrated a strong dependence on loading position.

Splinting may further explain the minimal differences in implant stress. Heo et al. [61] found that splinted implant-supported prostheses in fibula-reconstructed mandibles provided more uniform stress distribution and lower displacement than more segmented designs. Accordingly, the nearly identical implant stresses observed here suggest effective load sharing through the titanium bar, while length-dependent effects became more apparent in peri-implant bone and fixation hardware. Filonenko et al. [62] also showed that alveolar-level fibula positioning generally reduced bone stresses, confirming the importance of reconstruction geometry.

Most evidence concerning implant rehabilitation of fibula free flaps remains clinical, whereas dedicated FEA studies are comparatively few and generally examine isolated variables. Indeed, Heo et al. [61] highlighted the scarcity of biomechanical investigations specifically addressing implant-supported rehabilitation in reconstructed fibulae. Hocková et al. [25] reported 100% survival and 91.0% success for 27 short and extra-short implants placed in osseous microvascular free flaps, including six fibula free flaps, after up to 40 months. Their subsequent prospective study reported 97.1% survival and 88.2% success at 63 months across different osseous flap types. These findings support the clinical feasibility of reduced-length implants, while the present analysis provides complementary biomechanical evidence.

Several limitations should be acknowledged. The model was derived from a single patient, limiting generalizability. Only a single static postoperative loading condition with a unilateral bite force of 40 N was simulated. This condition represents the immediate postoperative phase and does not reproduce the progressive recovery of masticatory forces, cyclic mastication, parafunctional loading, or patient-specific bite-force variability. Consequently, the present model cannot predict fatigue-related failure of the fixation plates, cumulative peri-implant bone microdamage, or whether the relative biomechanical ranking of the implant lengths investigated would be maintained under higher functional loads. Although cortical bone was modeled as orthotropic, tissues were assumed homogeneous and linearly elastic, and bone remodeling, callus maturation, and progressive osseointegration were not simulated. The frictional implant-bone interface represents only an immediate-loading condition, while fully constrained condyles simplify temporomandibular joint behavior. Finally, only one implant diameter, placement scheme, and splinted prosthetic design were investigated. Therefore, these results should be interpreted as comparative biomechanical trends requiring validation in larger patient-specific and clinical studies.

## 5. Conclusions

Within the limitations of this patient-specific finite element study, implant length mainly affected peri-implant strain distribution and load transfer to the osteosynthesis system, while implant and fibular cortical bone stresses varied only slightly. The 5 mm configuration showed the lowest peri-implant strains and posterior plate stress, whereas the 12 mm implants produced greater apical strain concentrations and higher stress transfer to the fixation system. Under the specific anatomical and immediate postoperative loading conditions investigated, increasing implant length did not provide a clear biomechanical advantage, suggesting that short and extra-short implants may represent a potential option when fibular anatomy limits conventional implant placement.

## Figures and Tables

**Table 1:** Orthotropic and isotropic material properties assigned to the mandibular bone tissues, callus, and implants.

Material	Symphysis	Body	Angle	Ramus	Condyle	Coronoid	Fibula Cortical	Dentin	Mandible Trabecular	Fibula Trabecular	Callus	Ti6Al4V
E_1_ (MPa)	20,492	21,728	23,793	24,607	23,500	28,000	28,000	17,600	300	292	0.2	114,000
E_2_ (MPa)	16,350	17,828	19,014	18,357	17,850	17,500	17,700	17,600	300	292	0.2	114,000
E_3_ (MPa)	10,092	12,700	12,757	12,971	12,650	14,000	17,700	17,600	300	292	0.2	114,000
ν_12_	0.34	0.34	0.3	0.28	0.24	0.23	0.237	0.34	0.3	0.3	0.3	0.33
ν_23_	0.22	0.2	0.22	0.23	0.25	0.28	0.231	0.34	0.3	0.3	0.3	0.33
ν_13_	0.43	0.45	0.41	0.38	0.32	0.28	0.42	0.34	0.3	0.3	0.3	0.33
G_12_ (MPa)	6908	7450	7579	7407	7150	7150	4690	6567	115.4	112.3	-	44,000
G_23_ (MPa)	4825	5083	4986	5014	5150	5300	3600	6567	115.4	112.3	-	44,000
G_13_ (MPa)	5317	5533	5493	5386	5500	5750	4720	6567	115.4	112.3	-	44,000

## Data Availability

The original contributions presented in this study are included in the article. Further inquiries can be directed to the corresponding author.
